# The Clinical Value of the Widal Test for Enteric Fever

**Published:** 1898-04

**Authors:** 


### The Clinical Value of the Widal Test for Enteric Fever.

Dr. W. Gilman Thompson (*British Medical Journal*, December 18, 1897) gives the following conclusions: From the foregoing brief study of 503 cases of all kinds, in which the Widal test was applied, the following conclusions may be drawn:

1. While the test is positive in the large majority of cases of enteric fever, and negative in the great number of other diseases, there is a margin of error 11 to 12 per cent. on each side of what might be called the normal line, between cases in which the test fails where it ought to succeed, and succeeds where it ought to fail, in order to make it of real clinical value.

2. This total of 23 per cent. of possible error unfortunately includes just those cases in which there is the greatest doubt upon the purely clinical side.

3. As a genuine diagnostic aid, the test has about the value of the diazo in typhoid urine, or the study of leucocytosis in pneumonia, that is, it is confirmatory in connection with appropriate symptoms, but misleading if positive reliance be placed upon it.

4. The fact that an experienced bacteriologist is required to make the test is offset by the ease of transportation of specimens of dried blood, which long retain the power of reaction, and it is greatly to be hoped that further possible improvements in technique may place this most ingenious test upon a firmer practical basis than at present can be claimed for it.—*St. Louis Medical and Surgical Journal.*

